# Eating and Grooming Abilities Predict Outcomes in Patients with Early Middle Cerebral Infarction: A Retrospective Cohort Study

**DOI:** 10.1155/2020/1374527

**Published:** 2020-05-11

**Authors:** Yumi Suzuki, Sachiko Tsubakino, Hiromi Fujii

**Affiliations:** ^1^Department of Occupational Therapy, Yamagata Prefectural University of Health Sciences, 260 Kamiyanagi, Yamagata 990-2212, Japan; ^2^Division of Occupational Therapy, Yamagata City Hospital Saiseikan, 1-3-26 Nanukamachi, Yamagata 990-8533, Japan; ^3^Graduate School of Health Sciences, Yamagata Prefectural University of Health Sciences, 260 Kamiyanagi, Yamagata 990-2212, Japan

## Abstract

Patients with cerebrovascular disorders are often forced to rest, with early prognosis made by bedside examination. However, overloading, for example, talking for a long time, may worsen the condition. We hypothesized that activities of daily living (ADL) from the Functional Independence Measure (FIM) that were actually performed regularly are useful to predict prognosis. The present study was aimed at determining the predictive items related to predicting prognosis from the status of early motor paralysis and ADL in patients with acute middle cerebral artery (MCA) infarction. We examined 367 patients with MCA infarction for Brunnstrom recovery stage (BRS) and FIM within 4 days of admission and modified the Rankin Scale before onset and just before discharge. Logistic regression analysis was used to compare two groups of patients based on their postdischarge destination (Home/another hospital or facility). The logistic regression analysis showed the following: BRS Hand: odds ratio (OR) 1.641 (95% CI 1.642 (1.336–2.017), *p* < 0.001); FIM Grooming: OR 1.279 (95% CI 1.220–1.807, *p* < 0.001); and FIM Eating: OR 1.280 (95% CI 1.102–1.488, *p* < 0.001). On the other hand, the ROC analysis showed the ROC area for Eating to be 0.830 (95% CI 0.787–0.874), for Grooming to be 0.81 (95% CI 0.765–0.865), and for BRS Hand to be 0.805 (95% CI 0.760–0.851). The BRS Hand and FIM Eating and Grooming domains were identified as predictive factors using the following cutoff points: BRS Hand stage V and FIM scores of 5 for Eating and 4 for Grooming. The cutoff points for the BRS Hand and FIM Eating revealed that, at a minimum, such patients can use the nonaffected hand. The presence of cognitive dysfunction or dysphagia affects these domains. Therefore, these results suggested that Eating and Grooming are appropriate as evaluation items.

## 1. Introduction

Stroke is a common medical problem in many countries and is recognized as a major cause of disability in adults and elderly people [1, 2]. Cerebral infarction is one of the most common causes of disability or loss of activity in daily life, especially among older people [3]. It changes not only the lives of patients but also those of their families [4, 5]. In middle cerebral artery (MCA) infarction, in particular, many obstructions are caused by the fragility of the blood vessel structure [6]. Such arterial infarctions have symptoms that vary due to the complexity of the blood vessels and the size of the reflux area [7, 8].

MCA infarction is known to cause patient death or severe physical dysfunction [9, 10]; however, Japanese Registered Occupational Therapists (OTRs), who are in charge of rehabilitation of patients with acute cerebral infarction, have experienced many cases with only mild dysfunction. As these examples show, MCA infarcts vary greatly in the degree of injury depending on the degree of occlusion and the site of loss of the reflux zone.

Today's treatment advances allow patients with MCA infarction with mild symptoms to be discharged early.

The OTRs involved in the acute treatment of stroke are required to predict the functional prognosis of treated patients with MCA infarction from an early stage [11]. To achieve this at the Barnes-Jewish Hospital, the OTR will perform several functional evaluations as an initial assessment within a short period following the onset of MCA infarction [12]. Although the types of outcomes are different, their work is very similar to that of OTRs working in Japanese acute care hospitals.

It is also extremely important that they consider the potential burden on caregivers and the need for community support after the patient is discharged.

The ability to predict the willingness of a stroke patient to rehabilitate, and thus the outcome of rehabilitation, can be challenging in the early days of rehabilitation. Therefore, such evaluation items used for acute patients with MCA infarction should be concise and simple in order to minimize the burden imposed on patients.

Several studies have shown that functional status at admission, stroke severity, motor function, trunk movements, and cognitive function are the most important outcome predictors in stroke patients [13]. Among the tests used to estimate the prognosis of patients, the National Institutes of Health Stroke Scale is considered by some to be an effective one [14–16]. However, the inspection items are complicated. Moreover, a focus on certain items of activities of daily living (ADL), namely, the ability to wear clothes, the ability to move a wheelchair, and the presence or absence of urinary incontinence, has been reported to predict the prognosis of patients [17, 18].

There are reports that dependence in dressing and bathing also contributes to the long-term prognosis of patients [19]. However, these domains of ADL are often banned for early-onset patients who must rest. The reason for a mandatory rest period is to prevent the progression of cerebral edema and the recurrence of cerebral infarction and to suppress an increase in blood pressure as a result of the patient's own body movements. As a result, it is often difficult for the OTR to intervene directly in the patient's ADL.

In such cases, the OTR must make an assessment of the patient based on their actual observations of the patient's ADL. We considered that the potential for physical and mental damage from observing the patient at the evaluation stage is extremely small, even immediately following the onset of an infarction. Thus, we examined whether the future outcomes of patients could be predicted from the results of the Functional Independence Measure (FIM) performed soon after admission. The purpose of this study was to identify domains from the status of ADL and early motor paralysis that could predict prognosis in patients with early MCA infarction.

## 2. Materials and Methods

This was a retrospective cohort study that used data extracted from the medical records of occupational therapy patients.

### 2.1. Patients

The study was conducted at the Department of Rehabilitation, Okitama General Hospital, where stroke patients are referred from the Department of Neurosurgery. This survey study was approved by the Ethics Committee of the hospital on February 14, 2014. The presentation of the survey method and the notification to patients were posted on the bulletin board of the Rehabilitation Department of the hospital for 3 months. We accepted notifications of patient refusal to participate by telephone, email, and postal mail. The study was conducted in accordance with the principles of the Declaration of Helsinki (1964).

In Japan, stroke patients receive medical treatment for about 1 month in the acute period; thereafter, patients with severe disabilities change hospitals, except for those who need medical care due to clinical problems, for whom specialized rehabilitation is implemented.

The patients who were diagnosed with MCA infarction, admitted to our hospital's Department of Brain Surgery Ward, and prescribed rehabilitation between April 1, 2012, and March 31, 2014, were included in the study. Patients with any of the following criteria were excluded from the study: death during the study period, more than one episode of MCA infarction, multiple medical histories, severe complications, and other ongoing neurological disease or long-term confusion. We calculated the necessary number of samples with the condition that the number of samples in the smaller group was “independent variable ×10” or more when binarized with dependent variables in a logistic regression analysis. Specifically, the independent variables were set to 18 including 17 FIM items and BRS hand. Even for a small group of dependent variables, 180 samples were planned for collection.

### 2.2. Types of Evaluations

In accordance with the purpose of this study, we analyzed the first occupational therapy evaluation data of patients who received inhospital rehabilitation. Initial assessments were conducted within 4 days of the patient's admission. The reason for performing the evaluation within 4 days rather than immediately after admission was that the medical condition might progress during the acute phase of cerebral infarction even if treatment begins in the hospital. We anticipated that it would take as long as 4 days for the symptoms to stop progressing and stabilize [20]. The BRS and FIM surveyed determined that there was little change time by time, although there were changes day by day. Patients who seemed to have changed were closely examined and followed up in the medical records.

All patient evaluations were undertaken using the following three reliability, validity, and sensitivity measures that have been used previously in studies of poststroke recovery [21–23]: Brunnstrom recovery stage (BRS), FIM, and the modified Rankin Scale (mRS).

Assessments of patients and record reviews were performed by qualified OTRs working in the clinical setting. The statistical analysis was performed under the direction of an OTR dedicated to basic research.

The mRS was performed within the 4 days prior to discharge to determine the degree of patient care expected after discharge. The mRS before onset was also performed by interviewing patients. (In some patients with language disabilities, we interviewed the families about the mRS.)

The BRS is used to assess motor paralysis in stroke patients [24, 25] and includes evaluation of the upper limbs, hand, and lower limbs. Recovery is divided into six stages, from stage I, where no muscle contraction is observed, to stage VI, which is slightly inferior to the nonparalyzed side. In stage III of the Hand, the fingers of the whole hand cannot be held open; in stage IV, there may be a small range of semivoluntary finger extension and side pinch (which cannot be released by the thumb); however, the patients are unable to perform confrontation between the thumb and the little finger. In stage V, opposite pinch, tube grip, ball grip, and voluntary finger extension are added.

The FIM, a universally standardized ADL evaluation method [26], was performed at admission to measure the ADL that the patients were actually doing. The FIM has 18 evaluation items that include Eating and toileting (motor FIM) and language understanding and memory (cognitive FIM). Each of the evaluation items are graded according to the seven levels of assistance ranging from dependence to complete independence, with a maximum total score of 126 and a minimum score of 18. These are determined on the following basis: the higher the FIM score, the greater the independence of the patient's ADL, whereas the lower the FIM score, the heavier the care required.

The mRS is a test that infers the patient's own perception of the degree of care based on their answers to questions [27]. Scoring is based on a 6-level evaluation of 0–5 (if death occurs, it becomes a 7-level evaluation) as follows: 0 is asymptomatic, 1 is symptomatic but no serious disability, 2 is slight disability, 3 is moderate disability, 4 is moderately severe disability, 5 is severe disability, and 6 is death.

The patients were classified into two groups based on outcome destination: the patient's home or a close relative's home (Home) and a hospital or resident facility (Changed Hospital). All patients underwent a standard rehabilitation program at the hospital for 1 hour per day. With standard rehabilitation, rehabilitation is initially performed at the bedside until the condition stabilizes. OTRs provide eating training and how-to instructions for self-care on the bed. Once the patient's condition stabilizes, they are trained on how to keep sitting safely. Physical therapists deliver direct exercise training, while OTRs train patients in changing clothes, various activities, and so on. Patients then receive standing and walking training in physical therapy and ADL training in occupational therapy, to reduce the amount of assistance needed from others. After about 30 days, patients are discharged to their homes if they have the ability to recover there or are transferred to another hospital or other facility if they require more therapy. This judgment must be made by agreement between the hospital staff, patients, and their family members.

### 2.3. Statistical Analysis

The independent variables were chosen based on clinical grounds and previous studies that demonstrated each as valid independent predictors of outcome in stroke patients [12]. We chose age, sex, diagnosis, paralysis side, and BRS for the Hand; the BRS for the Upper Limb was strongly correlated with the Hand (*r* = 0.89) and thus could not be selected. We determined that the BRS for the Lower Limb was heavily loaded in early-onset patients. We checked the scatter plot in advance and confirmed that there were no variables that showed a significant linear relationship in the FIM items. For the FIM, we chose the following items as independent variables: Transfers-Bed, Grooming, Eating, Expression, and Comprehension.

The dependent variable was the outcome destination (Home or Changed Hospital). Eighteen patients (mRS score > 4) were treated before the onset. However, we decided that it would affect the initial ADL but not the purpose of the study and made no corrections.

We performed statistical processing using SPSS Ver23. First, the BRS item and the FIM item were compared between the Home and Changed Hospital groups using the Mann-Whitney *U* test and the chi-squared test. Next, BRS and FIM items were entered as independent variables with the outcome destination as the dependent variable, and binomial logistic regression analysis was performed by the stepwise method. The goodness-of-fit of the logistic regression was tested using receiver operating characteristic (ROC) curves and the Hosmer and Lemeshow test. We plotted an ROC curve to determine the cutoff values. The significance level was less than 5% (*p* < 0.05).

## 3. Results

**Figure 1:**
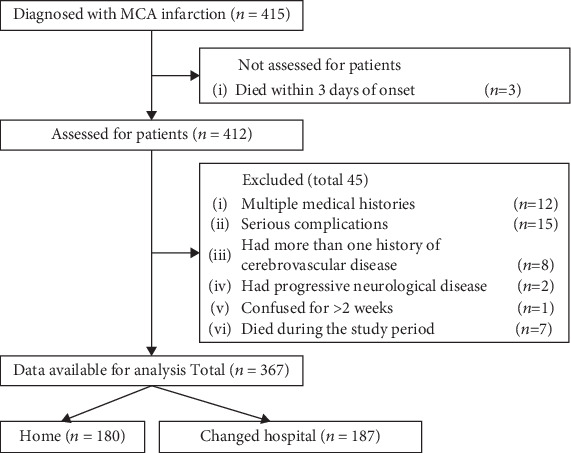
Flowchart of the study population.

During the study period, 415 patients were diagnosed with MCA infarction. Of these, three died within 3 days of onset and seven died during the study period. Twelve patients had multiple medical histories and 15 had serious complications. Eight patients had more than one history of cerebrovascular disease, two patients had progressive neurological disease, and one had been confused for more than 2 weeks. Patients with multiple medical histories had intracerebral hemorrhage, cerebral infarction, and femoral neck fracture; the serious complications were pneumonia, heart disease, and stomach cancer; the multiple cerebrovascular diseases were cerebellar infarction, intracerebral hemorrhage, and internal carotid artery infarction; and the progressive neurological disease was Parkinson's disease. After excluding these patients, there were 367 participants in the study (age 39–101 years, average age 78 years) (Figure 1).

Table 1 shows the demographic and clinical characteristics of the patient population. In the survey period of 2 years, there were 102 males and 78 females in the Home group and 85 males and 102 females in the Changed Hospital group. The duration of occupational therapy was 18 ± 14 days for patients who returned home and 33 ± 15 days for those who were moved to a hospital or other facility.

Before the onset of MCA infarction, there were 156 patients in the Home group and 136 patients in the Changed Hospital group who did not need care (Table 2).

Fifty-eight patients in the Home group had an mRS score of 0 before discharge, meaning they did not require nursing care. The remaining patients in the Home group needed some kind of care. In the Changed Hospital group, five patients had an mRS score of 0 before discharge; these patients anticipated further recovery or could not decide where to go. Most (182) of the patients in this group needed care; 28 patients had a score of 5, requiring severe care.

Table 3 shows the odds ratios (ORs) and 95% confidence interval (CI) from bivariate logistic regression analyses for independent variables. Of the 15 variables analyzed, three were significantly associated with the probability of outcome prioritization. The extracted variables were BRS for the Hand (OR 1.642, 95% CI 1.336–2.017, *p* < 0.001), Grooming (odds ratio 1.485, 95% CI 1.220–1.807, *p* < 0.001), Eating (OR 1.280, 95% CI 1.102–1.488, *p* < 0.001), and Age (OR 0.971, 95% CI 0.943–0.999, *p* < 0.045). The result of the model chi-squared test was significant (*p* < 0.01), and each variable was also significant (*p* < 0.010). The Hosmer and Lemeshow test result was *p* = 0.275, and the percentage of correct rate was 84.7%. The respective correlation coefficients were *r* = 0.585 (*p* < 0.001) between BRS Hand and FIM Eating, *r* = 0.541 (*p* < 0.001) between BRS Hand and FIM Grooming, and *r* = 0.658 (*p* < 0.001) between FIM Eating and Grooming.

**Figure 2:**
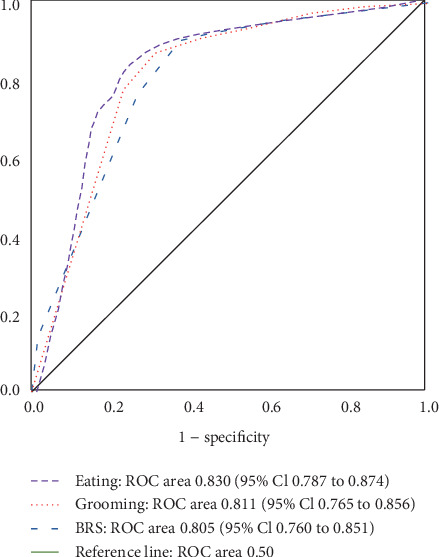
ROC curve of the BRS Hand, FIM Eating, and FIM Grooming items.

Figure 2 shows the area under the curve (AUC) in the ROC analysis of BRS Hand, Eating, and Grooming.

The AUC was ROC area 0.805 (95% Cl 0.760 to 0.851) in the BRS Hand, 0.830 (95% Cl 0.787 to 0.874) in Eating, and 0.811 (95% Cl 0.765 to 0.856) in Grooming.

**Figure 3:**
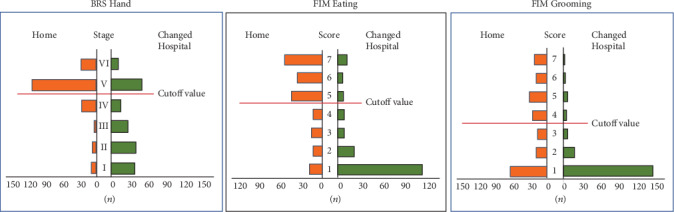
Results of the BRS Hand item staging and FIM scores for the Eating and Grooming domains in the Home and Changed Hospital study groups. The cutoff values are highlighted.

In the AUC for age, the ROC area was 0.632 (95% Cl 0.576–0.689), and the predictive ability was low. The cutoff values, which indicate patients identified with early MCA infarction who would return to their homes after discharge, were stage IV for the BRS Hand and FIM scores of 5 in Eating and 4 in Grooming (Figures 2 and 3).

Table 4 shows patients whose ADL items were determined as showing independence at BRS Hand stage V or higher. For the FIM Eating item, 47 people (about 30%) in the Home group and eight people (16%) in the Changed Hospital group were independent, while 27 people (19.6%) in the Home group and two people (4%) in the Changed Hospital group were independent for Grooming.

Model chi-squared test: *p* < 0.01. Correct classifications: 83.4%.

## 4. Discussion

The purpose of this study was to determine factors identifiable at the time of admission that are predictive of the outcome destination in inpatients with MCA infraction, based on the level of motor paralysis and a single FIM item. While there are patients with MCA infarction with conditions such as cardiogenic embolism that involve more serious symptoms, others are characterized by relatively mild symptoms and include lacunar infarction [28]. Nevertheless, in our study, 35 out of 88 patients with lacunar infarction had to change hospital. Our results showed that the FIM scores for Eating and Grooming items and BRS Hand staging within 4 days of admission were predictors of functional outcome.

Snickars et al. reported that the patient's finger extension 3 days after onset predicts upper limb function 1 month after a stroke [29]. In our study, the cutoff value for the BRS Hand on the 4th day after onset was stage V, which is almost comparable with the results reported by Snickars et al. BRS Hand stage V indicates the time when recovered finger separation and extension movements appear. Thus, the upper limb function of patients who have reached stage V early often shows good recovery.

In the Home group, 112 patients had a BRS Hand stage V on admission and 26 patients had a score of VI; among these, 27 patients had a FIM Grooming score of 7 and 47 patients had an Eating score of 7. Among patients with good BRS Hand function, only 30% were also independent for Eating while even fewer (20%) were independent for Grooming. In the Changed Hospital group, there were 12 BRS Hand stage IV patients and 50 stage V patients. From their initial ADL scores, eight patients from Eating and only two patients from Grooming had a score of 7. Moreover, there were only 26 patients who were transferred with an mRS score of 0 or 1.

These results showed that even if a patient's paralyzed hand has good finger function, it does not necessarily lead directly to ADL. We thought that the patient's paralyzed hand could not cope with the various movements of ADL. For example, brushing teeth requires changing hand movements with the sensation of a brush moving in the mouth. In addition, wearing T-shirts requires exploring invisible body parts using the hand. These may be regarded as advanced movements in which the motor and sensory functions of the hand cooperate.

ADL performance and upper limb function are expected to be closely related, but only a few associations have been clearly shown [30, 31]. Nevertheless, we included the BRS Hand in our analysis in part based on this conventional wisdom. About 77% of the Home group had a BRS Hand stage V or VI, even within 4 days of onset of infarction. This level of hand functioning involves full cooperation with the nonparalyzed hand in both hand movements. It is assumed that such functioning leads to an improvement in patients' ADL.

Nakao et al. reported that a Barthel Index score 3 weeks after onset is a predictor of ADL disorder 6 months later [32]. It is important to note that the Barthel Index assesses whether the patient can perform the activity at all, regardless of whether or not they perform it daily [33, 34]. Even if a patient is able to carry out even one item, the FIM can be evaluated at 7 levels, and the evaluation is highly reliable. Taub et al. reported that urinary incontinence is the best predictor of functional prognosis associated with ADL among patients with paralysis, urinary incontinence, speech disorder, and dysphagia within 24 hours after onset. [35]. Although incontinence is indeed an important factor in predicting outcomes, many patients also have urinary retention early after a stroke, and it is difficult to assess the presence or absence of incontinence. While dysphagia is also important in stroke patients, it is not included as an FIM item. If a patient has dysphagia, it is presumed that the FIM score may be lowered due to the need for assistance with eating and the time being lengthened by careful eating. If there is hemispatial neglect, which is one of the symptoms of MCA infarction, some assistance is required to ensure that the patient pays attention to the neglected side early after the onset. For example, caregivers must be vigilant about putting food on the nonattended side of the patient and ensuring that the patient does not spill hot food, etc.

There is no deduction item for hemispatial neglect in the FIM. However, by examining the patient's behavior, we can take into account the effects of unilateral neglect appearing in that behavior. A score of 5 on the FIM Eating item is the level at which patients can eat without assistance and implies that at least one hand can be used and that cognitive function has been maintained. The same is true for the Grooming item. Although Grooming is not directly required to support life, if it is unilaterally performed by another person, it can be an unpleasant activity for the patient. Therefore, patients unconsciously make attempts at Grooming, which is a sign of minimum self-care. In other words, the ability to seek a comfortable bodily sensation within 4 days of onset may indicate that the patients perceive a good prognosis for themselves. Grooming is also an important component of social awareness. Patients who show concern about their cleanliness shortly after onset are considered to have room for recovery.

The levels of ADL utilized in this study are not related to lower limb function. In addition, unlike other FIM items, no significant body movement is required. Common to the domains of Eating and Grooming is that each involves simple repetitive actions that can be completed with the use of just one hand [36]. However, it could be said that the use of a tool complicates an activity. Thus, while these tasks are simple, we propose that they are suitable to evaluate patient performance in the acute phase. The main limitation of this study is that it used patient data from a single department, whose post hospitalized treatment policies might differ from those of other institutions. Given the variations in treatment approaches for early cerebral infarction, studies at other facilities should be conducted. Moreover, although the deadline for the initial evaluation was limited to 4 days from the onset of MCA infarction, variations in the evaluation time of each patient during this early period could not be unified. We presume that by setting the time from the onset of the MCA infarction and converting the evaluation date and the evaluation time, more accurate data could be obtained.

Given that Eating and Grooming are ADL items that can be performed with minimal effort on the part of the patient, it is useful to be able to predict future outcomes from these items. This information would be useful not only for the OTR but also for anyone involved in patient care, such that a plan could be created for the patients' life activities.

## 5. Conclusions

Our study suggests that the BRS Hand stage V and the FIM domains of Eating (score 5) and Grooming (score 4) are effective at predicting the outcome of patients with acute MCA infarction.

## Figures and Tables

**Table 1:** Patient demographics and characteristics within 4 days after the onset of MCA infarction.

Variables	Total	Home	Changed hospital	*p* value
Number of subjects (*n*)	367	180	187	
Age, mean (SD)	76.6 ± 10.5	74.2 ± 10.4	78.9 ± 9.9	0.03
Men (*n*)	187	102	85	0.04
Women (*n*)	180	78	102	
Infarction type (*n*)				0.03
Cardiogenic embolism	154	63	91	
Atherosclerosis	110	56	54	
Lacunar	88	53	35	
Others	15	8	7	
Paralyzed side (*n*)				0.88
Right	192	103	89	
Double	3	2	1	
No paralysis	3	2	1	
BRS hand at onset (*n*)				0.01
I	78	8	70	
II	40	6	34	
III	16	3	13	
IV	45	25	20	
V	159	112	47	
VI	29	26	3	
FIM onset (score)				
Total	39 (18–126)	77 (18–126)	20 (18–114)	0.01
Length of hospital stay (days)	25.7 ± 16.1	18.2 ± 13.2	32.9 ± 14.9	0.01

**Table 2:** Comparison of between-group differences in mRS scores taken before the onset of MCA infarction and at the time of discharge.

mRS score	Before onset (*n*)^∗^		Discharge (*n*)^∗∗^	
	Home	Changed hospital	Home	Changed hospital
0	156	136	58	5
1	16	24	78	21
2	4	6	24	26
3	1	6	10	32
4	3	10	10	75
5	0	5	0	28

^∗^ *p* < 0.01, ^∗∗^*p* < 0.001.

**Table 3:** Binomial logistic analysis of domains predictive of hospital discharge.

Predictor	Partial regression coefficient	Odds ratio with 95% Cl	*p* value
BRS hand	0.496	1.642(1.336-2.017)	0.001
FIM grooming	0.395	1.485 (1.220-1.807)	0.001
FIM eating	0.247	1.280 (1.102-1.488)	0.001
Age	-0.030	0.971(0.943-0.999)	0.045
Constant	-1.498		0.205

**Table 4:** Comparison of the number of patients in each group who acquired a score of 7 for each FIM item among patients with a BRS score of V or higher for the Hand item.

Total (*n*)	Home 138		Changed hospital 50	
FIM item 7 score (*n*)	*n*	%	*N*	%
*Total score 126*	*2*	*1.4*	*0*	*0.0*
Eating	47	34.1	8	16.0
Grooming	27	19.6	2	4.0
Bathing	18	13.0	1	2.0
Dressing upper body	33	23.9	5	10.0
Dressing lower body	28	20.3	5	10.0
Lower toileting	24	17.4	3	6.0
Bladder control	27	19.6	3	6.0
Bowel control	31	22.5	3	6.0
Transfer to bed/chair/wheelchair	31	22.5	3	6.0
Transfer to toilet	26	18.8	2	4.0
Transfer tub/shower	11	8.0	1	2.0
Walk or wheelchair	23	16.7	4	8.0
Stairs	8	5.8	2	4.0
Comprehension	58	42.0	10	20.0
Expression	50	36.2	9	18.0
Social interaction	47	34.1	8	16.0
Problem solving	43	31.2	8	16.0
Memory	45	32.6	８	16.0

## Data Availability

The datasets used to support the findings of this study are available from the corresponding author upon request.
